# The Synergistic Effect of High Pressure CO_2_ and Nisin on Inactivation of *Bacillus subtilis* Spores in Aqueous Solutions

**DOI:** 10.3389/fmicb.2016.01507

**Published:** 2016-09-21

**Authors:** Lei Rao, Yongtao Wang, Fang Chen, Xiaojun Liao

**Affiliations:** ^1^Beijing Advanced Innovation Center for Food Nutrition and Human Health, College of Food Science and Nutritional Engineering, China Agricultural UniversityBeijing, China; ^2^Key Laboratory of Fruit and Vegetable Processing, Ministry of AgricultureBeijing, China

**Keywords:** high pressure CO_2_, nisin, synergistic inactivation, *Bacillus subtilis* spores, inner membrane damage

## Abstract

The inactivation effects of high pressure CO_2_ + nisin (simultaneous treatment of HPCD and nisin, HPCD + nisin), HPCD→nisin (HPCD was followed by nisin), and nisin→HPCD (nisin was followed by HPCD) treatments on *Bacillus subtilis* spores in aqueous solutions were compared. The spores were treated by HPCD at 6.5 or 20 MPa, 84–86°C and 0–30 min, and the concentration of nisin was 0.02%. Treated spores were examined for the viability, the permeability of inner membrane (IM) using flow cytometry method and pyridine-2, 6-dicarboxylic acid (DPA) release, and structural damage by transmission electron microscopy. A synergistic effect of HPCD + nisin treatment on inactivation of the spores was found, and the inactivation efficiency of the spores was HPCD + nisin > HPCD→nisin or nisin→HPCD. Moreover, HPCD + nisin caused higher IM permeability and DPA release of the spores than HPCD. A possible action mode of nisin-enhanced inactivation of the spores was suggested as that HPCD firstly damaged the coat and cortex of spores, and nisin penetrated into and acted on the IM of spores, which increased the damage to the IM of spores, and resulted in higher inactivation of the spores.

## Introduction

Bacterial endospores are metabolically dormant, and extremely resistant to the treatments such as heat, desiccation, UV, and γ-radiation, and some bactericidal chemicals because of their unique structures (Setlow, 1995, 2006; Setlow and Johnson, 2012). As spores of a number of *Bacillus* and *Clostridium* species are agents of food spoilage and food borne diseases (Brown, 2000; Logan, 2012; Setlow and Johnson, 2012), inactivation of spores has been receiving great attention in the food industry. Traditionally, thermal processing at relative high temperature (121°C or higher) is an efficient way to eliminate spores. However, the high temperature compromises organoleptic properties and causes some detrimental effects to the nutritional quality of heat-sensitive food. Consequently, there is a requirement for new ways of mild processing procedures to inactivate spores.

The inactivation effect of high pressure CO_2_ (HPCD) was firstly shown on *Escherichia coli* in 1951 (Fraser, 1951). In the recent years, a number of reports indicate that HPCD at pressure < 30 MPa and temperature of 20–40°C can effectively inactivate the vegetative forms of pathogenic and spoilage bacteria, yeasts, and molds (Spilimbergo and Bertucco, 2003; Zhang et al., 2006c; Perrut, 2012), and simultaneously maintain or improve the nutrient quality of liquid food (Damar and Balaban, 2006; Garcia-Gonzalez et al., 2007). Considering the efficient bactericidal effect of HPCD as well as its ability of maintaining or improving food quality, HPCD has been proposed as an alternative non-thermal pasteurization technique for foods. However, a problem of application HPCD in food processing is that HPCD at 20–40°C cannot inactivate bacterial spores (Rao et al., 2015a), which could be a potential risk for food safety (Brown, 2000). Generally high temperatures (>60°C) are needed for HPCD treatment to effectively inactivate spores (Enomoto et al., 1997; Ballestra and Cuq, 1998; Spilimbergo et al., 2003; Watanabe et al., 2003; Bae et al., 2009; Rao et al., 2015b). A variety of chemicals are reported to be combined with HPCD to increase the inactivation effect of spores, and hydrogen peroxide, tert-butyl hydroperoxide, peracetic acid, or trifluoroacetic acid could enhance the inactivation efficiency of spores by HPCD treatment at mild conditions (35–60°C) (White et al., 2006; Zhang et al., 2006a,b, 2007; Hemmer et al., 2007; Shieh et al., 2009; Tarafa et al., 2010; Checinska et al., 2011; Setlow et al., 2016). However, as the addition of these chemicals into food are prohibited, they cannot be applied in the sterilization procedures of food processing.

Nisin is an antimicrobial peptide produced by certain strains of *Lactococcus* (Delves-Broughton, 1990), and inhibits the growth of gram-positive bacteria and their endospores (de Arauz et al., 2009). It is generally regard as safe (GRAS) and its application in food has been approved by United States Food and Drug Administration (USFDA). Nisin acts on Gram-positive bacteria by forming pores in cell membranes (Ruhr and Sahl, 1985) or inhibits cell wall biosynthesis by disrupting of transglycosylation via binding to and mislocation of lipid II, a precursor for cell wall biogenesis (Wiedemann et al., 2001; Hasper et al., 2006). However, nisin shows no activity on Gram-negative bacteria because its access into the cytoplasmic membrane is blocked by the outer membrane (Stevens et al., 1991). In our previous study, Li et al. (2016) reported that HPCD + 0.02% nisin at 10 MPa and 32°C for 15 min showed an enhanced inactivation of *E. coli* in aqueous solutions compared with HPCD alone at the same condition, and this enhancement was due to the damage to the outer membrane of *E. coli* by HPCD, and then nisin penetrated into and act on the cytoplasmic membrane. Similar to the gram-negative bacteria, nisin also cannot act on the bacterial spores of dormant form because its access into the inner membrane (IM) is blocked by the coat and thick cortex of spores (Black et al., 2007; Gut et al., 2008). But after spore germination, the cortex and coat are degraded, and nisin can act on the IM by forming pores via binding to the lipid II, inhibiting the germinated spores outgrowing into vegetative cells (Gut et al., 2011). Given these findings, we assumed that nisin can theoretically act on the IM of the spores and inhibit their outgrowth if the out layers of spores including cortex and coat were damaged. Zhang et al. (2006b) reported that HPCD could damage the envelope of *Bacillus atrophaeus* spores inoculated on filter paper including exosporium, coat, cortex and IM through transmission electronic microscopy (TEM) images and DPA analysis. Meanwhile, our previous results also showed that HPCD at 20 MPa and 84–86°C kill the spores of *B. subtilis* in aqueous solutions most likely by destroying the structure of spores (Rao et al., 2015b). Thus, we assumed that nisin may act on HPCD-treated spores, and increase the inactivation of spores. In fact, a recent report indicated that combined treatment of HPCD at 30 MPa and 60°C for 120 min (4 cycles of 30 min each) and 0.01–0.5% nisin showed synergistic effect on inactivating *B. subtilis* and *Geobacillus stearothermophilus* spores inoculated on the surface of metal plates (Da Silva et al., 2016), but it did not show how this synergistic effect happened.

In this study, we investigated the effect of different combined treatments of HPCD and nisin on inactivating of *B. subtilis* spores in aqueous solutions, and figured out the role of the nisin in the inactivation of spores by HPCD. The IM damage of spores was analyzed by detecting the IM permeability using flow cytometry method (FCM) and DPA release, and the structural damage of spores was observed by TEM.

## Materials and Methods

### Strain and Spore Preparation

*Bacillus subtilis* 168 was obtained from China General Microbiological Culture Collection Center (Beijing, China). Overnight cultures of *Bacillus* strain grown in nutrient broth (Beijing Aoboxing Biological Technology Co. Ltd., Beijing, China) were transferred to sporulation agar plates, nutrient agar (Beijing Aoboxing Biological Technology Co. Ltd., Beijing, China) containing 50 μg/mL Mn^2+^. After 1 week incubation at 37°C, the spores were harvested in a sterile flask by flushing the surface of the culture with sterile distilled water and scrapping the surface with sterile glass microscope slide. The spores collected were washed three times by centrifugation at 7000 × *g* and 4°C for 15 min using a CF16RXII centrifuge (Hitachi, Japan), resuspended in sterile distilled water with a concentration of approximate 10^9^ CFU/mL, and stored at 4°C until they were used. All spores (>99%) used in this work were free of growing and sporulating cells, germinated spores and cell debris as determined with a BX45-72P15phase contrast microscope (Olympus, Japan). The concentration of the spore suspension was adjusted to approximate 10^7^ CFU/mL before treatments.

### HPCD and Nisin Combined Treatment

One gram of nisin (10^6^ IU/g) was dissolved in 50 mL distilled water and filter sterilized using 0.22 μm sterile filter. Then, the nisin solution was added to the spore suspensions before immediate HPCD treatment. The final concentration of the nisin was 0.002% (20 IU/mL), 0.01% (100 IU/mL), 0.02% (200 IU/mL), 0.04% (400 IU/mL), respectively. HPCD was performed with a batch HPCD system (Liao et al., 2007). For each experiment, 20 mL of the spore suspension with pH 6.5 were transferred to a 50 mL sterile glass tube and the tube was covered with a plastic film with a 0.22 μm membrane filter in the center of aeration to prevent microbial contamination. As the pressure vessel of the HPCD system reached the experimental temperature (84–86°C), the sample tubes were placed in the pressure vessel. Then, the vessel was pressurized by the plunger pump to 6.5 or 20 MPa within 0.1 min or 2.5 min, respectively. After holding for required treatment time, the depressurization was performed by opening the pressure relief valve at CO_2_ outlet on the pressure vessel. The depressurization time was 0.5 and 2.5 min for 6.5 and 20 MPa, respectively. After HPCD, the sample tubes were taken out from the vessel and analyzed immediately. The CO_2_ purity was 99.5% in all the experiment treatments. The combined treatments of HPCD and nisin was carried out as follows. (i) HPCD→nisin treatment: the spore suspensions without nisin were treated by HPCD at 20 MPa and 84–86°C for 30 min, then cooled down to ambient temperature, and 0.02% nisin was added into the HPCD-treated spores for 30 min, then centrifuged at 4°C and 7000 × *g* for 10 min and resuspended in sterile distilled water. (ii) nisin→HPCD treatment: the spore suspensions were treated with 0.02% nisin at ambient temperature for 30 min, centrifuged at 4°C and 7000 × *g* for 10 min and resuspended in sterile distilled water, then nisin-treated spores were treated by HPCD at 20 MPa and 84–86°C for 30 min. (iii) HPCD + nisin treatment: the spore suspensions were treated by HPCD and 0.02% nisin at 20 MPa and 84–86°C for 30 min, then cooled down to ambient temperature, centrifuged at 4°C and 7000 × *g* for 10 min and resuspended in sterile distilled water.

The inactivation of the spore suspensions by heat treatment with or without nisin at 86°C was carried out at 0.1 MPa without CO_2_ using a water bath. Twenty microliter of the spores suspended in sterile distilled water with pH 6.5 or pH 3.0 (HCl was used to adjust the acidity) was transferred to a 50 mL sterile glass tube, which was then immersed in a water bath equilibrated at 86°C for 0–30 min. After treatments, the sample tubes were taken out and analyzed immediately.

### Enumeration of Surviving Spores

The number of surviving spores was determined by the viable plate count method. For the spores treated with nisin, the samples were centrifuged at 4°C and 7000 × *g* for 10 min and resuspended in sterile water to eliminate the nisin. Then, each sample was serially (1:10) diluted with sterile distilled water and pour-plated on nutrient agar (Beijing Aoboxing Biological Technology Co. Ltd., Beijing, China) in duplicate. The plates were incubated at 37°C for 24 h. After incubation, the colonies were counted.

### Measurement of DPA

The DPA release was measured using the fluorescence method (Hindle and Hall, 1999). The treated spores were centrifuged at 7000 × *g* and 4°C for 10 min (CF16RXII, Hitachi, Japan), and assaying DPA in the supernatant solution was carried out by its fluorescence with Tb^3+^ in a 96-well plate. One hundred μL of supernatant solution were added to 100 μL 20 μmol/L terbium (III) chloride hexahydrate (99.9%, Aladdin Industrial Corporation, Shanghai, China) buffered with 1 mol/L acetic acid (99.8%, Beijing Chemical Works, Beijing, China) at pH 5.6. All the samples were analyzed with a Multiskan MK3 microplate reader (Thermo, MA, USA). Samples were excited at 270 nm, and emission spectra were collected at 545 nm. The total amount of DPA in each individual batch was determined after autoclaving at 121°C for 20 min (Zhang et al., 2006a), which was used as a positive control while the one in untreated spores was used as a negative control. HPCD-induced DPA release ratio was calculated by the equation as follows:

$$D\,P\,A\%=\frac{F_{1}-F_{0}}{F_{2}-F_{0}}\,\;\;\;\;\;\;\;\;\;\;\;\;\;\;\;\;\;\;\;\left(1\right)$$

Where *F*_0_, *F*_1,_ and *F*_2_ were the fluorescence intensity of untreated spores, HPCD-treated spores and autoclaved-spores, respectively.

### Flow Cytometry Analysis

Samples for flow cytometry were prepared with propidium iodide (PI), a DNA staining dye according to a reported method (Reineke et al., 2013). PI is membrane-impermeable and can be used to indicate the IM damage (Mathys et al., 2007). The treated spore suspensions were adjusted to concentrations of about 10^7^ spores/mL in sterile distilled water. The concentration of the PI was 15 μmol/L in the spore suspensions. Afterward, the samples were stored in the dark at room temperature for 45 min (Pappas et al., 2015).

Stained samples were then analyzed with an Accuri C6 (BD Accuri Cytometer Inc., USA) flow cytometry equipped with a 488 nm, 50 mWlaster. PI fluorescence was quantified with the FL2 detector at 585 ± 20 nm. The forward scatter threshold was set at 5000 to ensure that the small spores were not omitted as events. Spores were analyzed at a nominal flow rate of 14 μL/min, with a stream core diameter of 10 μm. All samples were evaluated after 30000 events had been recorded.

### Spores Preparation for TEM

Samples were prefixed in 2.5% glutaraldehyde (Sigma–Aldrich) overnight at room temperature, rinsed three times in 0.1 mol/L phosphate-buffered saline (PBS) for 15 min, then postfixed in 1% osmium tetroxide (Sigma–Aldrich) for 90 min and rinsed three times in 0.1 mol/L PBS for 15 min, and subsequently dehydrated with ethanol series. Afterward, the samples were embedded in epoxy resin and kept at 37°C overnight followed by 60°C for 24 h. The resin blocks were cut into ultrathin sections of 70 nm with a Lecia EM UC6 ultramicrotome (Leica, Germany) and stained with 3% aqueous lead citrate and uranyl acetate. Finally, the samples were examined by a H-7650B TEM (Hitachi, Japan).

### Data Analysis

Flow cytometry data were analyzed using the FlowJo version 7.6.1 software (FlowJo, OR, USA). Analysis of variance (ANOVA) was carried out by using software PASW statistic 18 (SPSS, USA). ANOVA tests were carried out for all experimental runs to determine significance at α = 0.05 level. All experiments were carried out in triplicate.

## Results

### Inactivation of Spores by Combined Treatment of HPCD and Nisin

**FIGURE 1:**
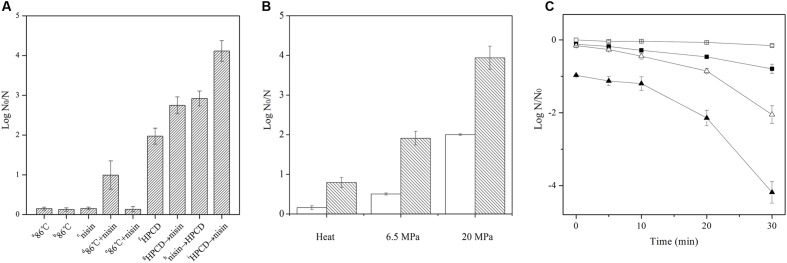
**Inactivation of *Bacillus sutilis* spores by (A) heat without or with nisin, or ordered sequential treatment of HPCD and nisin**. ^a^Spores in aqueous solutions with pH 6.5 were treated by heat at 0.1 MPa and 86°C for 30 min; ^b^Spores in aqueous solutions with pH 3.0 were treated by heat at 0.1 MPa and 86°C for 30 min; ^c^Spores in aqueous solutions with pH 6.5 were treated by 0.02% nisin at 0.1 MPa and ambient temperature for 30 min; ^d^Spores in aqueous solutions with pH 6.5 were treated by heat at 0.1 MPa and 86°C for 30 min with 0.02% nisin; ^e^Spores in aqueous solutions with pH 3.0 were treated by heat at 0.1 MPa and 86°C for 30 min with 0.02% nisin; ^f^Spores were treated by HPCD at 20 MPa and 84–86°C for 30 min; ^g^Spores in aqueous solutions with pH 6.5 were treated by HPCD at 20 MPa and 84–86°C for 30 min followed by 0.02% nisin treatment at 0.1 MPa and ambient temperature for 30 min; ^h^Spores in aqueous solutions with pH 6.5 were treated by 0.02% nisin at 0.1 MPa and ambient temperature for 30 min followed by HPCD treatment at 20 MPa and 84–86°C for 30 min; ^i^Spores in aqueous solutions with pH 6.5 were treated by HPCD at 20 MPa and 84–86°C for 30 min with 0.02% nisin; **(B)** heat or HPCD at different pressures without (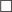) or with 0.02% nisin (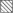). Heat: 0.1 MPa, 86°C, 30 min; HPCD: 6.5 MPa or 20 MPa, 84–86°C, 30 min; **(C)** heat or HPCD without (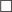, 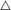) or with (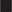, 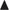) 0.02% nisin at different times. (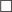, 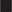) Heat: 0.1 MPa, 86°C, 30 min; (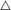, 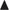) HPCD: 20 MPa, 84–86°C, 30 min.

In a preliminary trial of screening the concentrations of nisin (Supplementary Figure S1), 0.02% nisin was chosen to combine with HPCD to inactivate the spores in this study. As shown in **Figure 1A**, both heat treatment at 86°C for 30 min and 0.02% nisin at ambient temperature for 30 min exhibited small inactivation (≤ 0.2 log reduction), while HPCD at 20 MPa and 84–86°C for 30 min displayed high inactivation (2.1 log reduction), indicating that HPCD exhibited significantly higher inactivation than heat or nisin, which was similar to our previous results (Rao et al., 2015b). When nisin was added, the inactivation of the spores by heat treatment at pH 6.5 (0.99 log reduction) was enhanced, but there was no enhancement of inactivation for the heat treatment at pH 3.0 (0.13 log reduction), indicating that acidity was not enough to effectively inactivate spores. For HPCD treatment, the combined treatments of HPCD and nisin achieved a synergistic inactivation effect, and HPCD + nisin achieved higher inactivation (4.1 log reduction) than HPCD→nisin (2.7 log reduction) or nisin→HPCD (2.9 log reduction). As the HPCD + nisin was most efficiency to inactivate spores, it was employed in the following study. The inactivation of spores by HPCD + nisin as a function of the pressure was shown in **Figure 1B**. The addition of nisin increased 1.4 and 1.9 log more reduction of the spores than HPCD at 6.5 and 20 MPa, respectively, indicating that higher pressure achieved stronger synergistic inactivation effect of the spores. Inactivation kinetics of the spores by HPCD + nisin was shown in **Figure 1C**. For the spores treated by HPCD at 20 MPa and 84–86°C, the inactivation showed no difference from 0 to 10 min, and then increased with increasing the time, exhibiting a slow to fast inactivation pattern. When nisin was added, the synergistic inactivation effect also increased with increasing the time, and the inactivation kinetics of the spores also exhibited a slow to fast inactivation pattern.

### FCM Analysis

**FIGURE 2:**
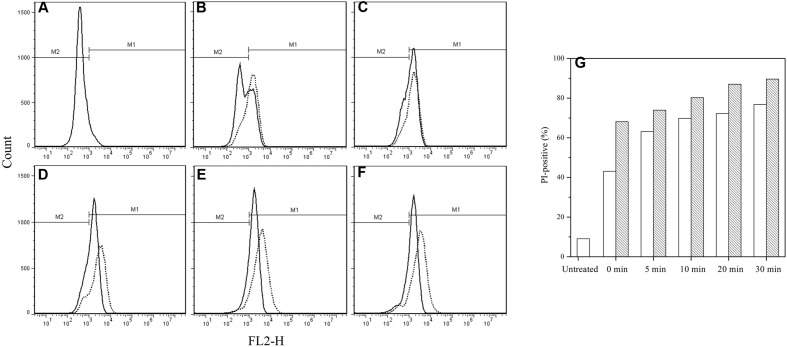
**Flow cytometry histograms of *B. subtilis* spores treated by HPCD at 20 MPa and 84–86°C for 0–30 min (solid line) and HPCD + nisin (dash line) using PI staining. (A)** untreated, **(B)** 0 min, **(C)** 5 min, **(D)** 10 min, **(E)** 20 min, **(F)** 30 min, **(G)** PI-positive percentage of (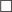) untreated or HPCD-treated spores and (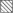) HPCD + nisin –treated spores, data obtained from flow cytometry histograms **(A**–**F)**.

The IM permeability of the spores treated by HPCD + nisin was examined by FCM with membrane-impermeable PI, and untreated spores were used as negative control. As shown in **Figure 2**, the FCM histograms of red fluorescence distribution of the spores stained by PI were divided into M1 and M2 regions. M2 was the negative area in which the spores were not stained by PI and had intact IM, while M1 was the positive area indicating that the spores were stained by PI and the IM of spores were damaged. Compared with the untreated spores (**Figure 2A**), the fluorescence distribution of the HPCD- and HPCD + nisin-treated spores were moved toward M1, but the HPCD + nisin-treated spores showed a stronger move than the HPCD-treated ones, indicating that HPCD + nisin achieved more severe damage to the IM of spores than HPCD. It was also evidenced by higher PI-positive percentage of the HPCD + nisin-treated spores, and the percentage was increased with increasing the treatment time (**Figure 2G**).

### DPA Release

**FIGURE 3:**
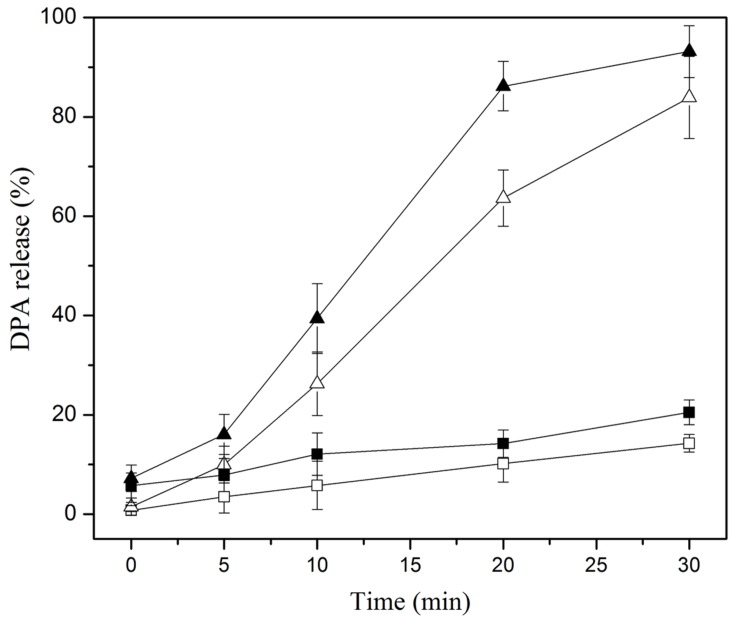
**DPA release of *Bacillus subtilis* spores treated by heat at 0.1 MPa and 86°C (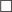) or HPCD at 20 MPa and 84–86°C (△), and heat + nisin (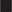) or HPCD + nisin (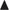)**.

The DPA release of the spores was another indicator of the damage to the IM of spores. As shown in **Figure 3**, the DPA release of the spores treated by heat at 86°C for 0–30 min was less than 14%, indicating that the IM of mostly heat-treated spores was intact. The DPA release of the spores was increased and less than 21% when the spores were subjected to heat + nisin, suggesting that the IM of many spores was still intact. The DPA release of the HPCD-treated spores for > 5 min was far higher than that by heat or heat + nisin, the highest DPA release was 80%, indicating the IM of mostly spores was damaged. Moreover, the DPA release of the HPCD + nisin-treated spores was significantly higher than that of the HPCD-treated spores, confirming that HPCD + nisin achieved more severe damage to the IM of spores.

## Discussion

In this study, the inactivation of the combined treatments of HPCD and nisin on *B. subitlis* spores in aqueous solutions was investigated, and a synergistic effect of HPCD + nisin, HPCD→nisin and nisin→HPCD on inactivation of spores was found (**Figure 1**). Similarly, Da Silva et al. (2016) reported that HPCD at 30 MPa and 60°C for 120 min was not able to efficiently inactivate (0.41 log reduction) *B. subtilis* spores inoculated on metal surface, while HPCD + 0.05% nisin achieved 3.2 log inactivation, showing an enhanced inactivation effect (**Table 1**). However, it not clear how nisin enhanced the inactivation of the spores by HPCD, and it was necessary to figure out possible action mode of nisin in the synergistic inactivation effect.

**Table 1:** Inactivation of wet or dry bacterial spores by HPCD treatment at different conditions.

	Strain species	Inoculating medium	Nisin (%)	HPCD treatment conditions			Log reduction	Reference
				Pressure (MPa)	Temperature (°C)	Time (min)		
Wet spores	*Bacillus subtilis*	Sterile water	0	20	84–86	30	1.97	This study
			0.02				4.11	
	
	*Bacillus subtilis*	Sterile water	0	6.5–25	86	60	>6	Rao et al., 2015b
	
	*Alicyclobacillus acidoterrestris*	Apple juice	0	10	70	30	>6	Bae et al., 2009

Dry spores	*Bacillus subtilis*	Sterile metal plates	0	30	60	120	0	Da Silva et al., 2016
			0.05				3.2	
			0.2				>7	
			3				>7	
	*Geobacillus stearothermophilus*		0				0	
			0.05				1.28	
			0.2				1.76	
			3				>5	
	
	*Bacillus anthracis*	Sterile filter paper	0.02^∗^	27.5	40	240	5.74–6.14	Zhang et al., 2007

*^∗^*Bacillus anthracis* spores were treated by HPCD and 0.02% hydrogen peroxide*.

**FIGURE 4:**
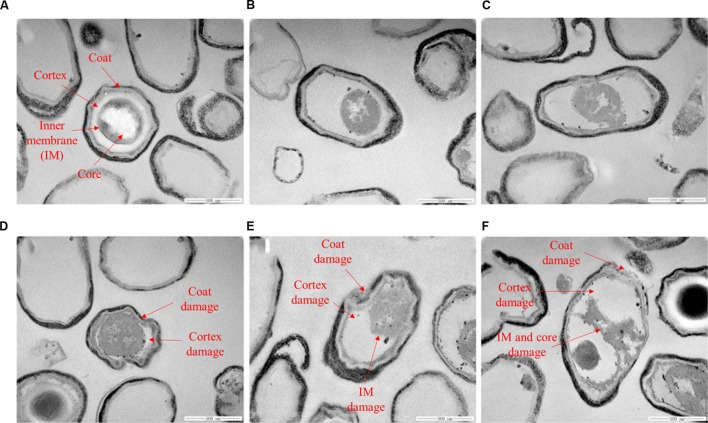
**Transmission electron microscopy images of untreated *B. subtilis* spores (A) or treated by HPCD at 20 MPa and 84–86°C for 0 min (B), 5 min (C), 10 min, (D), 20 min (E), 30 min (F)**.

**FIGURE 5:**
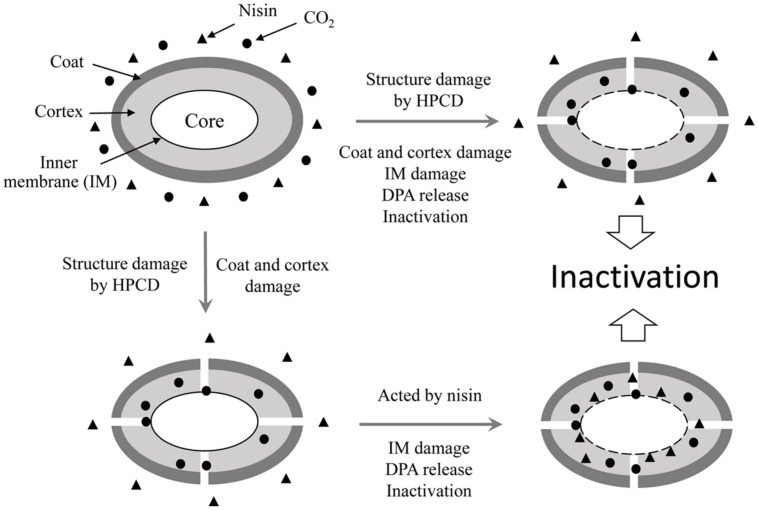
**Outline of the process of inactivation *B. sutilis* spores by HPCD + nisin**. During the process, structures of a partial number of spores including the coat, cortex and the inner membrane (IM) were damaged by HPCD, and these spores released DPA, lost resistance and were inactivated. Structures of another amount of spores including the coat and cortex were damaged, then nisin penetrated into the spore cells and acted on the IM, resulting in the damage to the IM and DPA release, which enhanced the inactivation of spores by HPCD.

It is reported that nisin cannot act on intact spores because its access to the IM was blocked by the coat and cortex, but after the spores germinated and degraded their coat and cortex, nisin could act on them and inhibit their outgrowth by forming pores in the IM of spores (Gut et al., 2011). Our previous work indicated that the inactivation of the spores in aqueous solutions by HPCD was likely attributed to the structural damage of the spores, rather than the germination (Rao et al., 2015b). Bae et al. (2009) observed damages to the surface and internal structures of *Alicyclobacillus acidoterrestris* spores in apple juice treated by HPCD at 10 MPa and 70°C for 30 min using scanning electron microscopy (SEM) and TEM (**Table 1**). Zhang et al. (2007) also evidenced that HPCD damaged the envelope of *B. atrophaeus* spores inoculated in filter paper including exosporium, coat, cortex, and IM (**Table 1**). In this study, the HPCD-treated spores showed visible structure changes with increasing the treatment time (**Figure 4**), especially after HPCD treatment of 10, 20, and 30 min, the damage to the coat (**Figures 4D–F**), cortex (**Figures 4D–F**), IM (**Figures 4E,F**), and core (**Figure 4F**) was manifested. These HPCD caused damages to the barrier (the coat and cortex of spores) blocking the access of nisin to the IM allowed nisin penetrated into and acted on the IM, and resulted in more damage to the IM of spores. This reasoning was evidenced by FCM (**Figure 2**) and DPA analysis (**Figure 3**), which suggested that the HPCD + nisin-treated spores exhibit higher IM permeability than the HPCD-treated spores. Therefore, we proposed that HPCD promoted the penetration of nisin into the spore cells by damaging the coat and cortex of spores, and then nisin acted on the IM by binding to the lipid II and forming pores in the IM, inhibiting the outgrowth of spores (Gut et al., 2011). As nisin-increased damage to the IM of spores (**Figures 2** and **3**) was coincident with the higher inactivation of the HPCD + nisin-treated spores (**Figure 1C**), the action mode of nisin in the synergistic inactivation effect of the spores was explained as follows (**Figure 5**). Firstly, HPCD damaged the coat and cortex of spores and increased their permeability, then nisin penetrated into the spores and acted on the IM by binding to the lipid II and forming pores, then increased more IM damage of the spores and resulted in higher inactivation of the spores. Similarly, the synergistic inactivation effect at higher pressures (**Figure 1B**) and prolonged times (**Figure 1C**) was also due to increased damage to the structure of spores, which benefited the penetration of nisin into the spores and increased the damage to the IM of spores and inactivation. Moreover, the enhanced inactivation efficiency of these HPCD and nisin treatments was HPCD + nisin > HPCD→nisin or nisin→HPCD (**Figure 1A**). Comparatively, HPCD→nisin generated lower synergistic inactivation effect of spores, and this was most likely attributed to the lower temperature during nisin treatment compared to HPCD + nisin treatment since higher temperature increased the efficiency of inactivating spores by nisin (**Figure 1A**). Meanwhile, nisin→HPCD also achieved lower synergistic inactivation effect of spores. Theoretically, nisin→HPCD should not enhance the inactivation of the spores since nisin at room temperature cannot act on the spores. Possible explanation for nisin→HPCD enhanced inactivation was probably due to the adherence of nisin to the surface of spores after nisin treatment (Chilton et al., 2013; Kraus et al., 2015), and the remaining nisin played an enhanced inactivation of the spores. Its action mode was similar to that of HPCD + nisin.

This study showed that HPCD + nisin achieved a synergistic inactivation effect of *B. subtilis* spores compared with HPCD or nisin alone, and this synergistic effect was due to nisin-increased damage to the IM of spores. Moreover, nisin could access into the IM of spores because HPCD damaged the barrier of spores including the coat and cortex. However, how HPCD damaged the barrier of spores is not clear, and needed to be further studied.

## Author Contributions

LR: carrying out the experiments and writing the manuscript. YW: giving advice and assistance for the experiment. FC: reviewing the manuscript and giving advice. XL: designing the experiment and revising the manuscript.

## Conflict of Interest Statement

The authors declare that the research was conducted in the absence of any commercial or financial relationships that could be construed as a potential conflict of interest.
