# Supplementary material for: The Synergistic Effect of High Pressure CO2 and Nisin on Inactivation of Bacillus subtilis Spores in Aqueous Solutions
Source: Front Microbiol. 2016 Sep 21;7:1507. doi: 10.3389/fmicb.2016.01507 (PMC5030830; doi:10.3389/fmicb.2016.01507)
Supplement: Supplementary file 1 [file Data_Sheet_1.DOCX]

Supplementary Material

**The synergistic effect of high pressure CO_2_ and nisin on inactivation of *Bacillus subtilis* spores in aqueous solutions**

***Lei Rao, Yongtao Wang, Fang Chen, Xiaojun Liao^^[[1]](#footnote-1)^*^***

*** Correspondence:** Corresponding Author: liaoxjun@hotmail.com

# Supplementary Figure


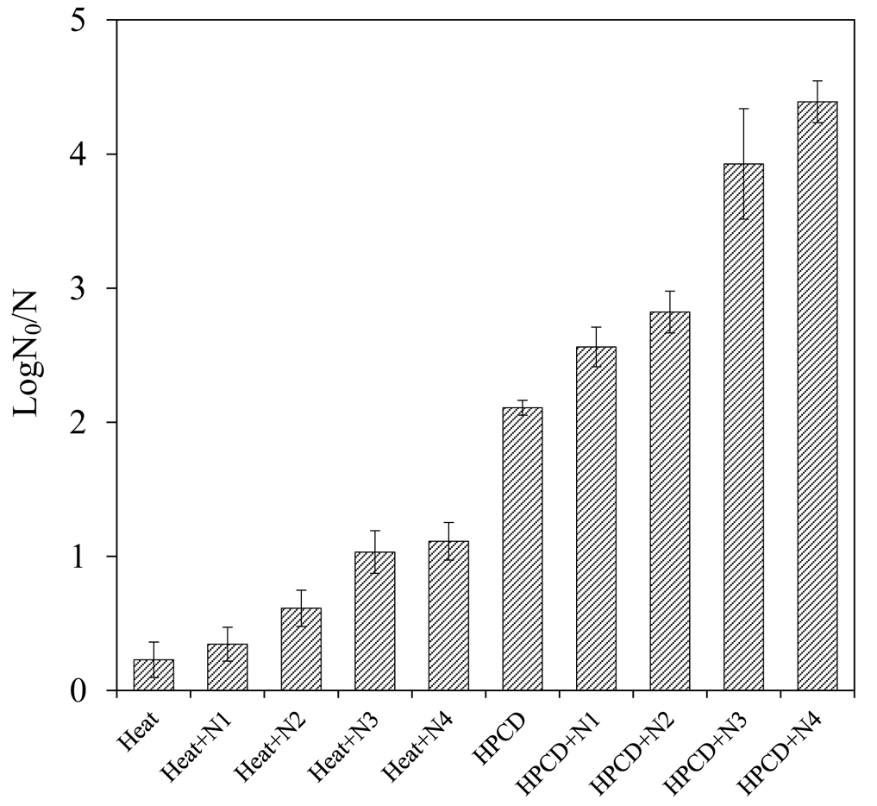


**Supplementary Figure 1.** Inactivation of *Bacillus sutilis* spores by heat or HPCD without or with different concentrations of nisin (N1-N4). Heat: 0.1 MPa, 86ºC, 30 min; HPCD: 20 MPa, 84-86ºC, 30 min; N1: 0.002%; N2: 0.01%; N3: 0.02%; N4: 0.04%.

1. *Corresponding Author. Fax: +0086-10-62737614; E-mail: liaoxjun@hotmail.com [↑](#footnote-ref-1)
